# Periodic Acid Modification of Chemical‐Bath Deposited SnO_2_ Electron Transport Layers for Perovskite Solar Cells and Mini Modules

**DOI:** 10.1002/advs.202300010

**Published:** 2023-05-04

**Authors:** Ziyi Wu, Jiazheng Su, Nianyao Chai, Siyang Cheng, Xuanyu Wang, Ziling Zhang, Xuanling Liu, Han Zhong, Jianfei Yang, Zhiping Wang, Jianbo Liu, Xin Li, Hong Lin

**Affiliations:** ^1^ State Key Laboratory of New Ceramics and Fine Processing School of Materials Science and Engineering Tsinghua University Beijing 100084 P. R. China; ^2^ State Key Laboratory of Advanced Technology for Materials Synthesis and Processing International School of Materials Science and Engineering Wuhan University of Technology Wuhan 430070 P. R. China; ^3^ Key Lab of Artificial Micro‐ and Nano‐Structures of Ministry of Education of China School of Physics and Technology Wuhan University Wuhan 430072 P. R. China; ^4^ Hubei Luojia Laboratory Wuhan 430072 P. R. China; ^5^ Wuhan Institute of Quantum Technology Wuhan 430206 P. R. China; ^6^ School of Microelectronics Wuhan University Wuhan 430072 P. R. China; ^7^ Key Laboratory of Advanced Materials of Ministry of Education of China School of Materials Science and Engineering Tsinghua University Beijing 100084 P. R. China; ^8^ School of Electronic Science and Engineering Xiamen University Xiamen 361005 P. R. China

**Keywords:** chemical bath deposition, large area, periodic acid, perovskite solar cells, SnO_2_

## Abstract

Chemical bath deposition (CBD) has been demonstrated as a remarkable technology to fabricate high‐quality SnO_2_ electron transport layer (ETL) for large‐area perovskite solar cells (PSCs). However, surface defects always exist on the SnO_2_ film coated by the CBD process, impairing the devices’ performance. Here, a facile periodic acid post‐treatment (PAPT) method is developed to modify the SnO_2_ layer. Periodic acid can react with hydroxyl groups on the surface of SnO_2_ films and oxidize Tin(II) oxide to Tin(IV) oxide. With the help of periodic acid, a better energy level alignment between the SnO_2_ and perovskite layers is achieved. In addition, the PAPT method inhibits interfacial nonradiative recombination and facilitates charge transportation. Such a multifunctional strategy enables to fabricate PSC with a champion power conversion efficiency (PCE) of 22.25%, which remains 93.32% of its initial efficiency after 3000 h without any encapsulation. Furthermore, 3 × 3 cm^2^ perovskite mini‐modules are presented, achieving a champion efficiency of 18.10%. All these results suggest that the PAPT method is promising for promoting the commercial application of large‐area PSCs.

## Graphical abstract

Chemical bath deposition (CBD) is extraordinarily suitable for depositing large‐area, dense and uniform SnO_2_ films, which is beneficial for the future commercialization of perovskite solar cells. A periodic acid post‐treatment is developed to modify the CBD‐SnO_2_ films. Periodic acid can not only react with the hydroxyl groups on the surface of SnO_2_ but also oxidize the Sn(II) to Sn(IV).

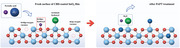

## 1 Introduction

Perovskite solar cells (PSCs) have made great progress over the past ten years,^[^ ^1^, ^2^, ^3^ ^]^ while the highest certified power conversion efficiency (PCE) exceeded 25% recently.^[^ ^4^ ^]^ A typical planar PSC device includes an electron transport layer (ETL), perovskite layer, hole transport layer (HTL), and electrodes. ETL plays a critical role in extracting and transporting the photogenerated electrons as well as blocking holes to reduce charge recombination.^[^ ^5^ ^]^ Therefore, the ETL's intrinsic optical and electronic properties will influence the photovoltaic performance and stability of a PSC. TiO_2_ is one of the most popular ETLs in PSCs due to its favorable electronic and optical properties. For a long period, the compact TiO_2_ (c‐TiO_2_)/mesoporous TiO_2_ (mp‐TiO_2_) stack occupies an important position in high‐efficiency PSCs.^[^ ^6^, ^7^ ^]^ However, TiO_2_ still has a few disadvantages, such as relatively low bulk electron mobility (<1 cm^2^ V^−1^ s^−1^), high processing temperature, and high photocatalytic activity, which may decrease the long‐term stability of PSCs.^[^ ^8^ ^]^ Recently, SnO_2_ has been considered as a promising ETL material owing to its outstanding bulk electron mobility (≈250 cm^2^ V^−1^ s^−1^), low‐temperature processability (<200 °C), and superb long‐term operational stability.^[^ ^9^ ^]^ Spin coating is the most typical technique to deposit SnO_2_ thin films. In 2015, Fang and co‐workers used solution‐processed SnO_2_ as ETL material of PSCs for the first time, achieving a champion PCE of 17.21%. They synthesized SnO_2_ films by a facile sol–gel approach: spin coating SnCl_2_·2H_2_O precursor solutions and followed by thermal annealing at 180 °C.^[^ ^10^ ^]^ Later, much work reported the deposition of synthesized SnO_2_ nanoparticles (NPs) or quantum dots (QDs) to form SnO_2_ films with better crystallization and less recombination centers.^[^ ^11^ ^]^ You and co‐workers used commercial SnO_2_ colloidal precursor solutions to fabricate a dense and pinhole‐free SnO_2_ film. The PSCs based on this low‐temperature solution‐processed SnO_2_ film achieved a certified PCE of 19.9%.^[^ ^12^ ^]^

Chemical bath deposition (CBD) is another good technique for fabricating SnO_2_ thin films. Different from the spin‐coating method, the CBD approach is extraordinarily suitable for depositing large‐area, dense and uniform SnO_2_ films without any area limits.^[^ ^13^ ^]^ It is very suitable for the future commercialization of perovskite solar cells. In 2016, Correa‐Baena and co‐workers firstly introduced the CBD technique for depositing the SnO_2_ layer in planar PSCs, which yielded a high open‐circuit voltage of 1.21 V at a bandgap of 1.62 eV, and achieved a stabilized PCE of 20.7%.^[^ ^14^ ^]^ Recently, Seo and co‐workers fabricated highly efficient PSCs based on CBD‐coated SnO_2_, with a certified PCE of 25.2%, indicating that CBD‐SnO_2_ could potentially improve photovoltaic performances.^[^ ^15^ ^]^ However, surface defects, such as oxygen vacancies and hydroxyl groups, always exist on the surface of CBD‐coated SnO_2_, which not only increase the non‐radiative recombination and impair the device's performances but also accelerate the degradation of the devices.^[^ ^16^ ^]^ Sargent and co‐workers reported surface modification of CBD‐SnO_2_ by ammonium fluoride, leading to reduced defects sites and better energy level match. This treatment achieved higher open circuit voltages and a champion PCE of 23.2%.^[^ ^17^ ^]^ Qi and co‐workers reported the incorporation of KMnO_4_ into the CBD process of SnO_2_ films. The strong oxidizing nature of KMnO_4_ promoted the conversion from Sn(II) to Sn(IV), leading to reduced trap defects and higher carrier mobility of SnO_2_. In addition, K^+^ ions and Mn^2+^ ions improved perovskite films’ crystallinity and phase stability.^[^ ^16^ ^]^

In this work, we developed a periodic acid post‐treatment (PAPT) method to modify the CBD‐SnO_2_ films. Periodic acid (H_5_IO_6_), with acidity and strong oxidizing properties, shows a bifunctional effect on SnO_2_ layers. It can not only react with the hydroxyl groups on the surface of SnO_2_ films but also oxidize the Tin(II) oxide (SnO) to Tin(IV) oxide (SnO_2_). With the help of periodic acid, a better energy level alignment between the SnO_2_ and perovskite layers and reduced defect sites in the SnO_2_ layer were achieved simultaneously. Furthermore, we investigated the effect of periodic acid on the photovoltaic performances and stability of the corresponding perovskite devices.

## 2 Results and Discussions

**Figure 1:**
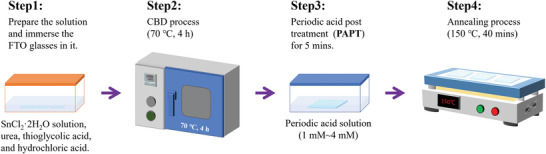
Schematic illustration of SnO_2_ films fabricated by the CBD process and PAPT method.

**Figure** **1** shows the schematic illustration of SnO_2_ films fabricated by the chemical bath deposition and PAPT method. Etched FTO glasses were immersed in dilute SnCl_2_ aqueous solution with urea, thioglycolic acid, and hydrochloric acid, and then heated at 70 °C for 4 h. For PAPT‐modified substrates, they were subsequently immersed in periodic acid solution with different concentrations for 5 min. After that, the substrates were thoroughly washed with deionized water to remove the residual reagents and annealed at 150 °C for 40 min.

**Figure 2:**
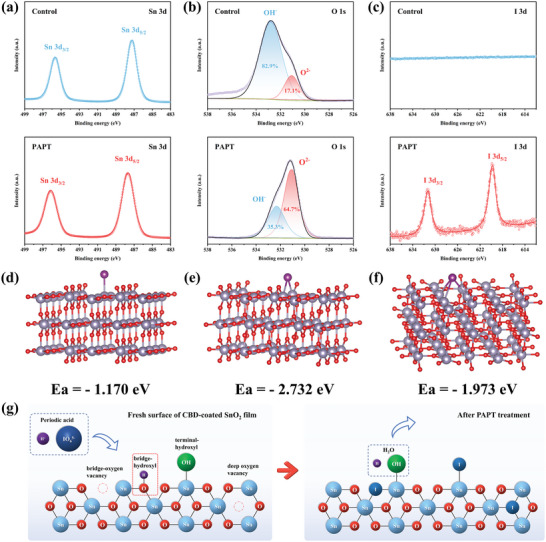
XPS spectra of a) Sn 3d, b) O 1s, and c) I 3d for the pristine SnO_2_ and PAPT‐modified SnO_2_ films. Hollow circle: raw data. Line: fitted data. All the SnO_2_ films were prepared via the CBD method. Absorption energy and corresponding structures. d) Iodine atom absorbed at the terminal Sn site. e) The iodine atom replaces bridge O site (iodine fills in the bridge oxygen vacancy). f) The iodine atom fills in the deep oxygen vacancy. g) Schematic image of the PAPT modification on the SnO_2_ surface.

To study the treatment mechanism of periodic acid to SnO_2_ films, we conducted X‐ray photoelectron spectroscopy (XPS) measurements. For the Sn 3d XPS spectra (**Figure** **2**a), the two peaks of Sn 3d_5/2_ and 3d_3/2_ in pristine SnO_2_ shifted from 487.2 and 495.6 eV to the higher binding energies of 487.6 and 496.1 eV, with the spin–orbit splitting energy of 8.4 eV.^[^ ^18^ ^]^ The higher binding energy of Sn 3d indicates more Sn (II)’s conversion to Sn (IV),^[^ ^16^, ^19^ ^]^ potentially widening the band gap and decreasing the number of oxygen vacancies.^[^ ^20^, ^21^ ^]^ In terms of O 1s spectra (Figure 2b), two peaks of around 532.5 and 531.1 eV refer to the hydroxyl group (–OH) on the SnO_2_ surface and the saturated lattice oxygen in SnO_2_ film, respectively.^[^ ^17^, ^20^ ^]^ The calculated areas of the –OH in pristine SnO_2_ reduced from 82.9% to 35.3% in the PAPT‐modified SnO_2_, indicating more chemisorbed non‐lattice oxygen transferred to lattice oxygen and existed in the form of SnO_2_. It is known that surface –OH introduces a deep energy level into the bandgap that is near the valence band, bringing the generation of non‐radiative recombination and energy losses in the devices.^[^ ^22^ ^]^ As shown in Figure 2c, two typical peaks of I 3d orbitals appeared in the PAPT‐modified SnO_2_ sample. In contrast, the pristine SnO_2_ sample showed no signal, indicating iodide remained on the PAPT‐modified SnO_2_ surface by chemical doping. The two peaks of I 3d_5/2_ and 3d_3/2_ located at 619.8 and 631.3 eV refer to iodine anion (I^−^), which were consistent with the referenced SnI_2_ signals in the previous reports.^[^ ^23^, ^24^ ^]^ According to the XPS results of Sn 3d and O 1s, we believe that the PAPT treatment urges the transformation of Sn(II) to Sn(IV) and the conversion of chemically absorbed hydroxyl to lattice oxygen, which lead to reduced trap states and potentially higher *V*_OC_ and FF of the PSCs.

As previously reported,^[^ ^17^, ^22^ ^]^ four kinds of oxygen defects exist on the surface of the CBD‐SnO_2_ films, including two kinds of hydroxyl defects and two kinds of oxygen vacancy defects (*V*_O_): 1) terminal hydroxyl (OH_T_), which is bonded to a single Sn atom on the surface (Figure S1a, Supporting Information); 2) bridge hydroxyl (OH_B_), including a bridge oxygen and an absorbed hydrogen atom (Figure S1b, Supporting Information); 3) bridge‐oxygen vacancy (*V*_OB_), i.e., the missing of the bridge oxygen atom (Figure S1c, Supporting Information); 4) deep oxygen vacancy (*V*_OD_), i.e., the missing of another oxygen atom located at a deeper layer than bridge oxygen (Figure S1d, Supporting Information). To figure out if the iodine atom will remain at the hydroxyl site or fill the *V*_O_, we conducted density functional theory (DFT) calculations. The stoichiometric (110) lattice plane was chosen as the studied surface due to that it is the most thermodynamically stable configuration.^[^ ^12^, ^25^ ^]^ According to the results of DFT calculations, the absorption energies (*E*_a_) of the iodine atom absorbed on terminal Sn (Figure 2d) and bridge O site (Figure 2e) are −1.170 and −2.732 eV. The negative value of *E*_a_ indicates that the iodine atom is prone to be absorbed in the same site after the hydroxyl groups were eliminated by periodic acid. Also, the more negative *E*_a_ implies iodine atoms are more apt to be absorbed in the bridge oxygen sites. As for *V*_O_, the *E*_a_ of the iodine atom substituted the bridge *V*_OB_ is exactly the same as previously calculated results, which is −2.732 eV. And the *E*_a_ of the iodine atom substituted at *V*_OD_ (−1.973 eV) showed a similar result (Figure 2f), indicating that iodine atoms can effectively passivate the *V*_O_ defects on the SnO_2_ surface. Above all, the schematic image of the PAPT method is shown in Figure 2g.

From the above analysis, we found that periodic acid has the following three functions: 1) H^+^ can decrease the surface hydroxyl groups; 2) The strong oxidation of ${\mathrm{IO}}_{6}^{5-}$ can oxidize Sn^2+^ into Sn^4+^; 3) The reduced I^−^ can also reduce the surface –OH and fill in the oxygen vacancies. To figure out which one plays a more critical role, we treated SnO_2_ films with sulfuric acid (H_2_SO_4_) and hydroiodic acid (HI), respectively. It should be noted that the concentrations of H_2_SO_4_ and HI are 5 and 2 mm, respectively, which are consistent with the H^+^ and I^−^ concentrations in the optimal concentration of periodic acid (2 mm, which will be discussed later).

XPS spectra of SnO_2_ films treated by H_2_SO_4_ and HI are shown in Figure S2 (Supporting Information). The double peaks of the Sn 3d of both samples are located at 487.2 and 495.6 eV (Figure S2a, Supporting Information), which is consistent with the position of the control sample (Figure 2a), indicating that a small amount of Sn^2+^ may exist in the SnO_2_ films. It is because dilute H_2_SO_4_ and HI cannot oxidize Sn^2+^ into Sn^4+^. As shown in Figure S2b (Supporting Information), the O 1s peak can be divided into two peaks corresponding to –OH and lattice oxygen, respectively. It can be seen that the –OH peak area of H_2_SO_4_‐modified SnO_2_ samples reduces from 82.9% to 40.1%, while that of HI‐modified samples only reduces it to 53.2%, indicating that H^+^ is more effective for eliminating the surface –OH than I^−^. Finally, we observed weak I 3d double peaks on the surface of the HI‐treated sample (Figure S2c, Supporting Information), the intensity of which is much lower than that of the H_5_IO_6_‐treated sample, suggesting that more iodine atoms in periodic acid stay on the SnO_2_ films. Above all, periodic acid has a stronger ability to eliminate surface oxygen defects than H_2_SO_4_ and HI. Furthermore, we carried out XPS depth analysis measurements to study the oxygen defects inside the SnO_2_ film. The etching depth is around 10 nm. It can be seen that the peak of O 1s is located around 531.1 eV (Figure S3, Supporting Information), corresponding to the position of lattice oxygen, while no –OH peaks are observed. In addition, I 3d double peaks are observed in PAPT‐modified SnO_2_ samples, indicating that only the periodic acid can fill the oxygen vacancies inside the SnO_2_ films.

**Figure 3:**
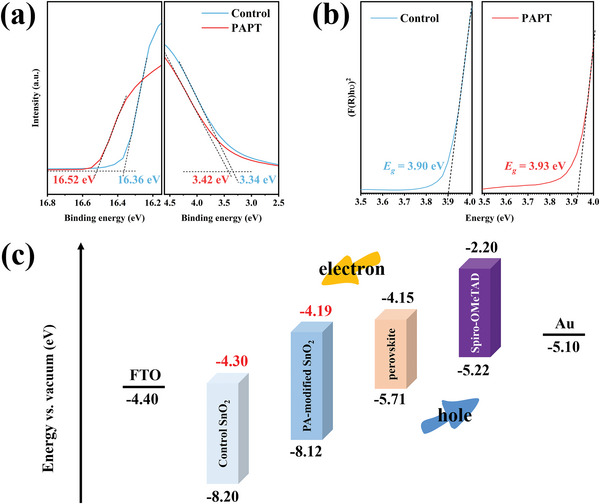
a) UPS spectra and b) UV–vis reflection spectra of the pristine SnO_2_ and PAPT‐modified SnO_2_ films. c) Schematic energy level diagram of FTO, the pristine SnO_2_, PAPT‐modified SnO_2_, and perovskite.

Ultraviolet photoelectron spectroscopy (UPS) measurements were performed to investigate the influence of energy level by periodic acid. The valence band maximum (VBM) values of samples can be calculated by the formula of VBM = 21.22 eV − *E*_cutoff_ + *E*_onset_.^[^ ^26^ ^]^ As shown in **Figure** **3**a, the corresponding VBM values of pristine SnO_2_ and PAPT‐modified SnO_2_ are 8.20 and 8.12 eV, respectively. In combination with the optical bandgap values of samples (Figure 3b), the conduction band minimum (CBM) values of pristine SnO_2_ and PAPT‐modified SnO_2_ are 4.30 and 4.19 eV, respectively. Similarly, the perovskite's CBM and optical bandgap can be calculated as 4.15 eV (Figure S4, Supporting Information) and 1.56 eV (Figure S5, Supporting Information), respectively. Therefore, the schematic energy level diagram of FTO, the pristine SnO_2_, PAPT‐modified SnO_2_, and perovskite is shown in Figure 3c. A better energy band alignment between the PAPT‐modified SnO_2_ and perovskite layers was achieved due to the upshift of the conduction band and Fermi level of SnO_2_. As a result, a reduced energy barrier and faster electron extraction at the SnO_2_ and perovskite interface could be realized.^[^ ^27^ ^]^

**Figure 4:**
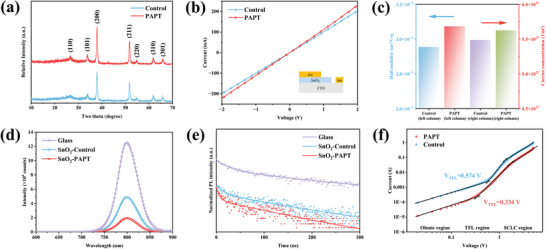
a) Grazing incidence X‐ray diffraction (GIXRD) spectra of the pristine SnO_2_ and PAPT‐modified SnO_2_ films. b) *I–V* curves of the devices based on the FTO/SnO_2_/Au structure for evaluating the conductivity of the pristine SnO_2_ and PAPT‐modified SnO_2_ films. c) Hall mobility and carrier concentration results of the pristine SnO_2_ and PAPT‐modified SnO_2_ films. d) Steady‐state PL spectra and e) Time‐resolved PL (TRPL) spectra of the perovskite films on glass, the pristine SnO_2_ and PAPT‐modified SnO_2_ films. f) Dark *J*–*V* characteristics of the electron‐only devices based on different SnO_2_ layer. (The device structure: FTO/SnO_2_/perovskite/PCBM/Ag).

Grazing incidence X‐ray diffraction (GIXRD) was conducted to investigate the change in the crystal structure of the SnO_2_ films. As shown in **Figure** **4**a, several peaks of the PAPT‐modified SnO_2_ film are slightly higher than that of the pristine sample, indicating a better crystallinity was obtained after PAPT treatment. We carried out electron conductivity and space‐charge‐limited current (SCLC) measurements of devices based on the FTO/SnO_2_/Au structure, as shown in Figure 4b and Figure S6 (Supporting Information). The PAPT‐modified SnO_2_ film exhibit higher conductivity and electron mobility than those of the pristine SnO_2_ film, suggesting the PAPT treatment could improve the electron transport of the SnO_2_ films. As shown in Figure 4c, Hall effect measurements demonstrate a similar result. The PAPT‐modified SnO_2_ film showed a higher hall mobility *µ*_H_ (3.07 × 10^−3^ m^2^ V^−1^ s^−1^) and larger carrier concentration *n* (5.63 × 10^27^ m^−3^) than those of the pristine SnO_2_ film (*µ*_H_ = 2.88 × 10^−3^ m^2^ V^−1^ s^−1^ and *n* = 5.36 × 10^27^ m^−3^).

According to the UV–visible light transmittance spectra of SnO_2_ films (Figure S7, Supporting Information), the PAPT treatment would not influence the high visible light transmission of SnO_2_ films. Atomic force microscopy (AFM) measurements were carried out to investigate the surface morphology of the chemical‐bath deposited SnO_2_ layers (Figure S8, Supporting Information). The PAPT‐modified SnO_2_ film showed a relatively lower roughness with a root mean square (RMS) of 9.91 nm compared to the pristine SnO_2_ film (RMS = 11.08 nm), indicating the PAPT treatment would not impair the crystal growth process of the upper‐level perovskite layers. Scanning electron microscope (SEM) of perovskite films (Figure S9, Supporting Information) deposited on the surface of the pristine and PAPT‐modified SnO_2_ films could also prove this point of view.

Steady‐state photoluminescence (PL) and time‐resolved PL (TRPL) measurements were carried out to study the charge transfer dynamics at the SnO_2_/perovskite interfaces. As shown in Figure 4d, the perovskite film on the PAPT‐modified SnO_2_ film exhibited only 23.1% PL intensity of that of the perovskite on the pristine SnO_2_ film. It indicates more efficient charge extraction of PAPT‐modified SnO_2_ film, owing to the better energy level match. For the TRPL spectra, both samples showed bi‐exponential decay curves (Figure 4e). The average carrier lifetime (*τ*_avg_) was fitted and listed in Table S1 (Supporting Information). The perovskite film deposited on the PAPT‐modified SnO_2_ film exhibited the *τ*_avg_ of 50.55 ns, which is about 60% shorter than that of the perovskite film on the pristine SnO_2_ film, 82.70 ns. It also suggests faster electron collection and transportation.

We also estimated the trap density by measuring the dark current density–voltage (*J–V*) curves based on electron‐only devices (FTO/SnO_2_/perovskite/PCBM/Ag). The typical dark *J*–*V* curves can be divided into three regions: ohmic region, trap‐filling limited (TFL) region, and trap‐free space charge limited current region. The electron defect density (*N*_t_) can be estimated by the trap‐filling limit voltage (*V*_TFL_).^[^ ^25^, ^28^ ^]^ The electron‐only devices based on the PAPT‐modified SnO_2_ film obtained a much lower *V*_TFL_ (0.334 V) and *N*_t_ (3.51 × 10^15^ cm^−3^) compared to those based on the pristine SnO_2_ film (*V*_TFL_ = 0.574 V and *N*_t_ = 6.04 × 10^15^ cm^−3^), as shown in Figure 4f. The fitting parameters (*R* ^2^) were listed in Table S2 (Supporting Information). Obviously, the PAPT treatment helps reduce the electron defect density and facilitates the charge transport between the SnO_2_ and perovskite films.

**Figure 5:**
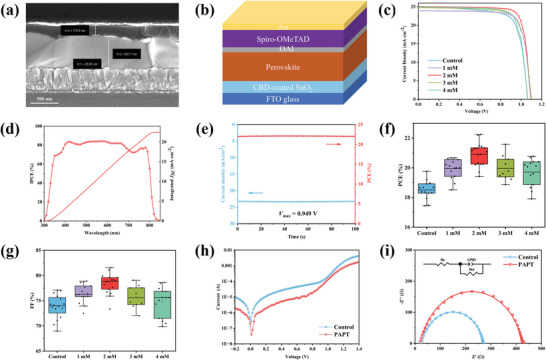
a) The cross‐section SEM image of the fabricated PSCs. b) The structure schematic image of a typical PSC device. (The device structure: FTO/PAPT‐modified SnO_2_/perovskite/OAI/Spiro‐OMeTAD/Au). c) *J–V* curves of the champion PSCs based on the different amounts of periodic acid treatments. d) EQE spectra for the PSCs based on the PAPT‐modified SnO_2_ film. e) Steady‐state photocurrent measurement of the champion PSC based on the PAPT‐modified SnO_2_ film. The applied bias voltage was 0.949 V, which was the voltage at the maximum power point. f) PCE and g) FF statistic distributions of the PSCs with different amounts of periodic acid treatments. h) Dark *I*–*V* curves of the PSCs based on the pristine SnO_2_ and PAPT‐modified SnO_2_ films. i) Nyquist plots of the PSCs based on the pristine SnO_2_ and PAPT‐modified SnO_2_ films, which was measured at a bias potential of 1.0 V in the frequency range of 1 MHz to 0.1 Hz in the dark.

To investigate the photovoltaic performances of PSCs with the PAPT‐modified SnO_2_ films, we fabricated devices with a structure of FTO/SnO_2_/perovskite/OAI/Spiro‐OMeTAD/Au, as shown in **Figure** **5**a,b. After careful optimization of the periodic acid concentration (1–4 mm) (Table S3, Supporting Information), an optimal concentration of the PAPT treatment (2 mm) led to a champion PCE of 22.25% (Figure 5c), with a *J*_SC_, *V*_OC_, and FF of 25.02 mA cm^−2^, 1.09 V, and 81.55%, respectively. Meanwhile, PSCs based on the pristine SnO_2_ layer only achieved a champion PCE of 19.77% with a *J*_SC_, *V*_OC_, and FF of 25.09 mA cm^−2^, 1.06 V, and 74.28%, respectively. The external quantum efficiency (EQE) spectrum of PSCs based on the PAPT‐modified SnO_2_ film led to an integrated *J*_SC_ of 22.45 mA cm^−2^, as shown in Figure 5d. The steady‐state PCE of the champion PSC is measured to be 22.10% under a constant bias voltage of 0.949 V (Figure 5e). Statistic distributions of the PCE, FF, and *V*_OC_ of the PSCs based on different concentrations of periodic acid are shown in Figure 5f,g and Figure S10 (Supporting Information).

The PCE enhancement is mainly attributed to the increment of FF and *V*_OC_, which benefited from the interface modification by the periodic acid treatment. In addition, we investigated the role of periodic acid on the transport properties in PSCs by dark *I*‐*V* characterization. As shown in Figure 5h, PAPT‐modified devices exhibited a significantly lower dark current, indicating the reduction of leakage current between the SnO_2_/perovskite interface, which could be attributed to the higher compactness and uniformity of the PAPT‐modified SnO_2_ films.^[^ ^25^ ^]^ Moreover, we conducted electrochemical impedance spectroscopy (EIS) to investigate the charge transfer and recombination processes in the PSCs. Figure 5i shows the Nyquist plots of devices based on the pristine SnO_2_ and PAPT‐modified SnO_2_ films, measured at a bias potential of −1.0 V under dark conditions. It is worth mentioning that the semicircle at the high‐frequency region representing the charge transfer resistance (*R*_ct_) was too small to be identified.^[^ ^29^ ^]^ Therefore, only one semicircle at the low‐frequency region was observed from the Nyquist plots, reflecting the interfacial recombination resistance (*R*_rec_) between the SnO_2_ layer and the perovskite layer. According to the fitting results (Table S4, Supporting Information) employing the equivalent circuit (inset of Figure 5h), the *R*_rec_ value increased from 240.6 to 399.7 Ω after the PAPT modification. The higher *R*_rec_ indicates a lower charge recombination rate,^[^ ^29^ ^]^ thus contributing to the improved FF and *V*_OC_. In addition, the fitted series resistance (*R*_S_) values are 29.49 and 23.96 Ω for PSCs based on the pristine SnO_2_ and PAPT‐modified SnO_2_ films, respectively. The smaller *R*_S_ is ascribed to increased conductivity of the SnO_2_ layer, consistent with the previous measurement. We also performed the electric capacity–voltage (*C–V*) measurement to analyze the built‐in potential (*V*_bi_) by the Mott–Schottky equation. As shown in Figure S11 (Supporting Information), the PAPT‐modified device displayed a higher *V*_bi_ (0.94 V) than the control device (0.77 V), indicating more efficient charge separation and collection and, thus, higher *V*_OC_.^[^ ^30^ ^]^ Drive‐level capacitance profiling (DLCP) measurement was conducted to obtain spatial distributions of trap densities in SnO_2_ films.^[^ ^31^, ^32^ ^]^ As shown in Figure S12 (Supporting Information), the trap density in PAPT‐SnO_2_ is relatively lower than that of the control‐SnO_2_ film, indicating PAPT modification is beneficial for reducing the defect density in the SnO_2_ layer. All these characterization results suggest the PAPT modification optimizes the interfacial charge transportation and inhibits the interfacial recombination between the ETL and perovskite layers, leading to better photovoltaic performances.

**Figure 6:**
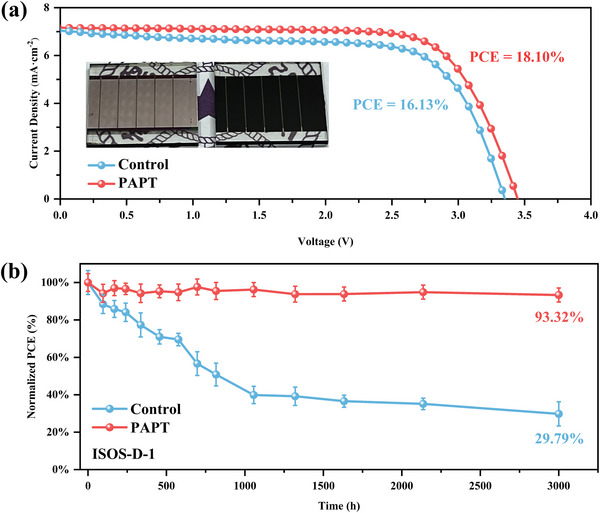
a) The photograph and the of the *J*–*V* curves of 3 × 3 cm^2^ modules based on control and PAPT‐modified SnO_2_ films. b) Stability tests of PSCs stored in 20 °C, 20–25% RH for 3000 h (ISOS‐D‐1 procedure).

Finally, we fabricated the laser‐etched perovskite solar mini‐modules (PSMs) with an area of 3 × 3 cm^2^ using a slot‐die coating method, as shown in **Figure** **6**a. The champion PSM based on the PAPT‐modified SnO_2_ film achieved an excellent PCE of 18.10% (*J*_SC_ = 7.16 mA cm^−2^, *V*_OC_ = 3.45 V, and FF = 73.42%) with an aperture area of 2.29 cm^2^, while the control one only achieved a PCE of 16.13% (*J*_SC_ = 7.08 mA cm^−2^, *V*_OC_ = 3.35 V, and FF = 68.02%). These results reveal that the CBD process with the periodic acid treatment is quite suitable for large‐area production of PSCs and PSMs. Next, we evaluated the stabilities of PSCs according to the International Summit on Organic PV Stability (ISOS) protocols.^[^ ^33^ ^]^ As shown in Figure 6b, the unencapsulated device based on the PAPT‐modified SnO_2_ film remained at 93.32% of its initial PCE after 3000 h of exposure to a relative humidity of 20–25% at 20 °C in an ambient atmosphere (conform to ISOS‐D‐1 procedure). The control device only kept 29.79% of its original PCE under the same conditions. The excellent stability of PAPT‐modified devices could be ascribed to the more compact and smoother interface between the SnO_2_ and perovskite films, with fewer defects and channels for moisture.

## 3 Conclusions

In summary, we developed a powerful periodic acid post‐treatment (PAPT) method to modify the SnO_2_ films fabricated by the CBD process. According to XPS results, it is found that periodic acid promoted the transformation of Sn(II) to Sn(IV) and the conversion of chemically absorbed hydroxyl to lattice oxygen, which could lead to reduced trap states and potentially higher *V*_OC_ of the PSCs. In addition, a better energy band alignment between the PAPT‐modified SnO_2_ and perovskite layers was achieved due to the upshift of the conduction band and Fermi level of SnO_2_. As a result, a reduced energy barrier and faster electron extraction at the SnO_2_ and perovskite interface could be realized. PSCs based on PAPT‐modified SnO_2_ layer achieved a champion PCE of 22.25%, with *J*_SC_, *V*_OC_, and FF of 25.02 mA cm^−2^, 1.09 V, 81.55%, respectively. Meanwhile, PSCs based on pristine SnO_2_ layer only achieved a champion PCE of 19.77% with *J*_SC_, *V*_OC_, and FF of 25.09 mA cm^−2^, 1.06 V, 74.28%, respectively. The PCE enhancement is mainly attributed to the increment of FF and *V*_OC_, benefited from the interface modification by the periodic acid treatment. In addition, the devices based on PAPT‐modified SnO_2_ exhibited excellent stability, which remain 93.32% of its initial efficiency stored at 20 °C, 20–25% RH after 3000 h without any encapsulation. Furthermore, we fabricated 3 × 3 cm^2^ perovskite mini‐modules based on PAPT‐modified SnO_2_ layers, achieving a high efficiency of 18.10% with the aperture area of 2.29 cm^2^, while the control one only achieved PCE of 16.13%. These results indicate that PAPT method efficiently boost the large‐scale production of SnO_2_ ETL and perovskite modules.

## Conflict of Interest

The authors declare no conflict of interest.

## Author Contributions

Z.W. and J.S. conceived the project, fabricated, and characterized the perovskite solar cells. N.C. provided support for the CBD process. X.W., Z.Z., X.L., H.Z., and J.Y. assisted to analyze and discuss the experiments results. J.L. contributed to the DFT calculations. S.C. and Z.W. contributed to CV and DLCP measurements. Z.W. wrote the manuscript. X.L. and H.L. supervised the work and revised the manuscript. All the authors discussed the results and commented on the manuscript.

## Supporting information

Supporting Information

## Data Availability

The data that support the findings of this study are available in the Supporting Information of this article.
