# Supplementary material for: Periodic Acid Modification of Chemical‐Bath Deposited SnO2 Electron Transport Layers for Perovskite Solar Cells and Mini Modules
Source: Adv Sci (Weinh). 2023 May 4;10(20):2300010. doi: 10.1002/advs.202300010 (PMC10369290; doi:10.1002/advs.202300010)
Supplement: Supplementary file 1 — Supporting Information [file ADVS-10-2300010-s001.pdf]

ADVANCED SCIENCE
Open Access

Supporting Information

for *Adv. Sci.*, DOI 10.1002/advs.202300010

Periodic Acid Modification of Chemical-Bath Deposited SnO_2 Electron Transport Layers for Perovskite Solar Cells and Mini Modules

Ziyi Wu, Jiazheng Su, Nianyao Chai, Siyang Cheng, Xuanyu Wang, Ziling Zhang, Xuanling Liu, Han Zhong, Jianfei Yang, Zhiping Wang, Jianbo Liu, Xin Li and Hong Lin**

Periodic Acid Modification of Chemical-bath Deposited SnO_2 Electron Transport Layers for Perovskite Solar Cells and Mini Modules

Ziyi Wu[1], Jiazheng Su[1], Nianyao Chai[2], Siyang Cheng[3, 4, 5, 6], Xuanyu Wang[1], Ziling Zhang[1], Xuanling Liu[1], Han Zhong[1], Jianfei Yang[1], Zhiping Wang[3, 4, 5, 6], Jianbo Liu[7], Xin Li[8, *], Hong Lin[1, *]

1 State Key Laboratory of New Ceramics and Fine Processing, School of Materials Science and Engineering, Tsinghua University, Beijing 100084, P.R. China

2 State Key Laboratory of Advanced Technology for Materials Synthesis and Processing, International School of Materials Science and Engineering, Wuhan University of Technology, Wuhan 430070, P.R. China

3 Key Lab of Artificial Micro- and Nano-Structures of Ministry of Education of China School of Physics and Technology, Wuhan University, Wuhan 430072, P.R. China

4 Hubei Luojia Laboratory, Wuhan 430072, P.R. China

5 Wuhan Institute of Quantum Technology, Wuhan 430206, P.R. China.

6 School of Microelectronics, Wuhan University, Wuhan 430072, P.R. China.

7 Key Laboratory of Advanced Materials of Ministry of Education of China, School of Materials Science and Engineering, Tsinghua University, Beijing 100084, P.R. China

8 School of Electronic Science and Engineering, Xiamen University, Xiamen 361005, P.R. China

* Corresponding author: lixin01@xmu.edu.cn and hong-lin@tsinghua.edu.cn

Materials

All the chemical reagents were directly used without further purification. Cesium iodide (99.999%), lead bromide ($PbBr_2$, 99.999%), Tin(II) chloride dihydrate (>99.995%), N,N-Dimethylformamide (DMF, anhydrous, 99.8%), dimethyl sulfoxide (DMSO, anhydrous, >99.9%), chlorobenzene (anhydrous, 99.8%), acetonitrile (anhydrous, 99.8%), bis(trifluoromethane) sulfonamide lithium salt (Li-TFSI, 99.95%), and 4-*tert*-butyl pyridine (t-BP, 98%), urea (99.0-100.5%) were purchased from Sigma-Aldrich. Formamidinium iodide (FAI, >99.99%) and methylammonium

iodide (MAI, >99.99%) were purchased from Greatcell Solar. Lead iodide (PbI_2, 99.99%), and thioglycolic acid (>95.0%) were purchased from Tokyo Chemical Industry (TCI). Periodic acid (99%) was purchased from Macklin. Hydrochloric acid (HCl), ethanol, and 2-propanol were purchased from Beijing TongGuang Fine Chemicals Company. FTO glass (12-14 Ohm/□) was purchased from Advanced Election Technology Co., Ltd. Methylamine chloride (MACl, 99.5%) was purchased from Xi'an Polymer Light Technology Co., Ltd. Spiro-OMeTAD (99.5%) and FK209 Co(III) TFSI salt (>99%) were purchased from Lumtec.

Preparation of SnO_2 layer by chemical bathing deposition (CBD)

The CBD solution was prepared by dissolving 0.5 g urea in 40 mL deionized water, followed by the addition of 10 μL thioglycolic acid and 500 μL HCl (37 wt%). After sonication for 5 minutes, 0.11 g $SnCl_2·2H_2O$ (99.995%) was dissolved in the CBD solution. The solution was stored at 8~10 °C for 72 h before use. 20 mL CBD solution was diluted with 100 mL de-ionized water, followed by sonication for 5 minutes. The temperature during the sonication process needs to be carefully controlled below 30 °C to prevent the occurrence of the chemical reaction.

FTO glasses were etched and cleaned in detergent, deionized water, acetone, ethanol, and 2-propanol. Before use, the substrates were dried with N_2 and exposed to an O_2 plasma atmosphere for more than 20 min. As-cleaned FTO glasses were horizontally placed in the CBD solution, followed by a heating process at 70 °C for 4 h. The FTO glasses were cleaned with deionized water 3~4 times. For the PAPT-modified samples, the FTO glasses were immersed in periodic acid solutions with different concentrations varied from 1 mM to 4 mM for 5 minutes. All the FTO glasses were dried with N_2 and annealed on a hot plate at 150 °C for 40 minutes.

Fabrication of perovskite solar cells

1.45M perovskite precursor solution (CsI: FAI: MAI: PbI_2: $PbBr_2$: MACl=:0.05: 0.81: 0.04: 0.98: 0.02: 0.20) were dissolved in DMF: DMSO=4: 1 solvent, as previously reported.[1] The precursor solution was spin-coated on the FTO substrate with a SnO_2 layer at 1000 rpm for 12 s and 5000 rpm for 28s. 160 μL chlorobenzene was dropped at 10s before the end of the second spinning process, followed by

annealing at 100 °C for 10 min and 150 °C for 10 min. The Spiro-OMeTAD solution was prepared by dissolving 0.1260 g Spiro-OMeTAD, 32 μL Li-TFSI solution (520 mg/mL in acetonitrile), 14 μL Co-TFSI (375 mg/mL in acetonitrile), 55 μL t-BP in 1.4 mL chlorobenzene. The spiro-OMeTAD solution was spin-coated on the perovskite film at 4000 rpm for 30 s. Finally, a 70 nm thick gold electrode was deposited by thermal evaporation.

Fabrication of perovskite solar modules (PSMs) via slot-die coating:

For the fabrication of PSMs, FTO substrates with areas of 3×3 cm^2 were first etched with a 1024-nm CO_2 laser (P1 scribing). Then, the SnO_2 films were fabricated by the CBD method. The slot-die machine was purchased from DaZheng (Jiangsu) Micro-Nano Tech. Co., Ltd. (China). Then, the perovskite precursor ink was diluted to a molar concentration of 0.8 M and slot-die coated on the substrates accompanied by a gas-quenching process (with a substrate-moving speed of 2 mm/s). The blowing gas pressure from the gas knife was 0.1 MPa. After that, the substrate was transferred onto a hot plate and annealed at 150 °C for 10 mins. Then, the Spiro-OMeTAD solution was slot-died coated at a speed of 2 mm/s. P2-scribe was conducted using a 532-nm-laser. Then, the P2-scribed substrate was evaporated with a mixed top electrode of MoO_x (5 nm)/ copper (100 nm). Afterward, the module was scribed again to complete the series connection using the same laser scriber. The ratio of the dead-area width to the active-area width was 1:9 (0.05mm to 0.45 mm), leading to a GFF of 90%.

Computational methods

First-principle calculations were performed with the density functional theory (DFT) as implemented in Vienna ab-initio Simulation Package (VASP) package.[2] The augmented wave (PAW) method[3, 4] was used for the interactions between ions and valence electrons with Perdew-Burke-Ernzerhof (PBE)[5, 6] functional. The plane wave energy cutoff was set to 450 eV. A sufficiently large vacuum layer of 15 Å was used to avoid the periodic image interactions along the z-direction. The atomic force and energy convergence criteria thresholds were set to 0.02 eV and 1×10−5 eV/ Å, respectively. The k-point mesh of 3×3×1 was employed for SnO2 surface geometric

optimization and adsorption energy calculation. The van der Waals (vdW) interaction was taken into account with the Grimme type at the DFT-D3 level.[7]

Characterization

The morphology of the perovskite films was observed by field-emission scanning electron microscopy (FESEM, LEO1530, Zeiss, Germany) and atomic force microscopy (AFM, Cypher, Oxford Instruments, UK). The crystal structures were characterized by X-ray diffraction (XRD, D8 Advance Diffractometer, Bruker, Germany) with Cu Kα (1.5406 Å) radiation operating at 40 kV. The Ultraviolet-Visible light spectrum was obtained using a UV-Vis spectrophotometer (Lambda 1050, Perkin Elmer, USA). The photoluminescence (PL) spectra were characterized by a fluorescence spectrometry (FLS920, Edinburgh Instruments, UK). For steady-state PL spectra, the spectra were obtained by illuminating the sample with a monochromatic xenon lamp source (λ_{exc}=460 nm). For time-resolved PL (TRPL), the spectra were acquired with samples photoexcited by a pulsed laser beam (405 nm). The XPS spectra were recorded by an X-ray photoelectron spectrometer (ESCALAB 250Xi, Thermo Fisher SCIENTIFIC INC., UK) with Al Kα radiation (hν = 1486.6 eV) as the illumination source. The photoelectron spectroscopy in the air (PESA) was performed with the AC-2 instrument (AC-2, RIKEN Instruments, Japan). The Fourier transform infrared spectroscopy (FTIR) measurements were performed on the infrared spectrometer (VERTEX 70V, Bruker, Germany) in ATR (Attenuated Total Reflection) mode. The photocurrent density-voltage (*J-V*) curves were obtained by a digital source meter (2400, Keithley Instruments, USA). The perovskite solar cells were illuminated under AM 1.5G (100 mW/cm^2) by solar simulators (91192 and 94082A, Newport, USA) calibrated with a standard crystalline silicon solar cell. The monochromatic incident photon-to-electron conversion efficiency (IPCE) spectra were obtained by the quantum efficiency system (QEX10, PV measurements, USA) in a DC mode.

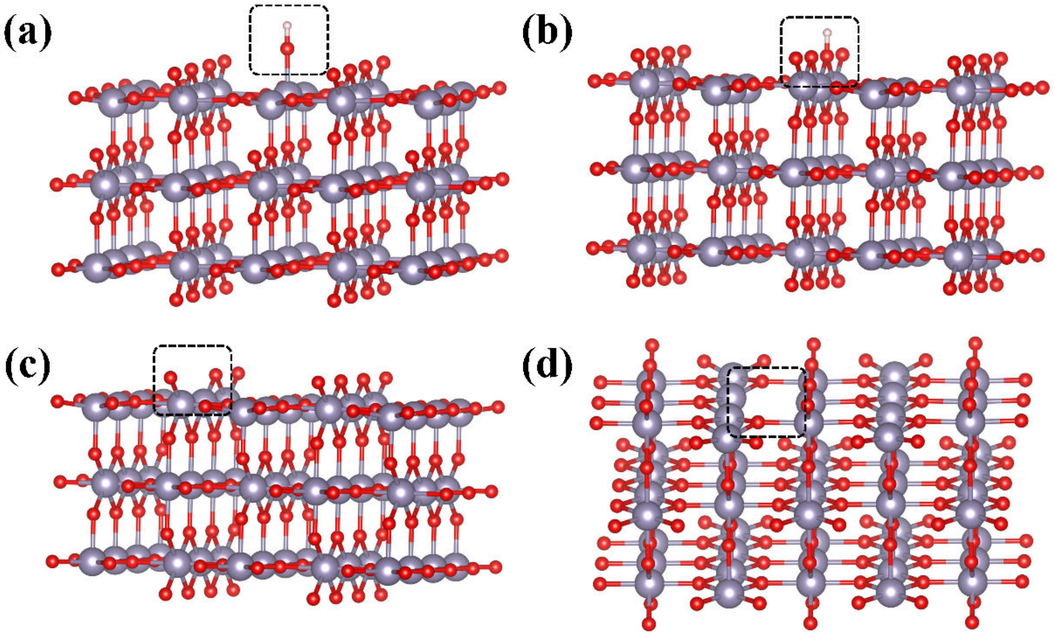

Figure S1 The structure image of four kinds of oxygen defects on the surface of CBD-coated SnO_2 films: (a) terminal hydroxyl (b) bridge hydroxyl (c) bridge-oxygen vacancy (d) deep oxygen vacancy.

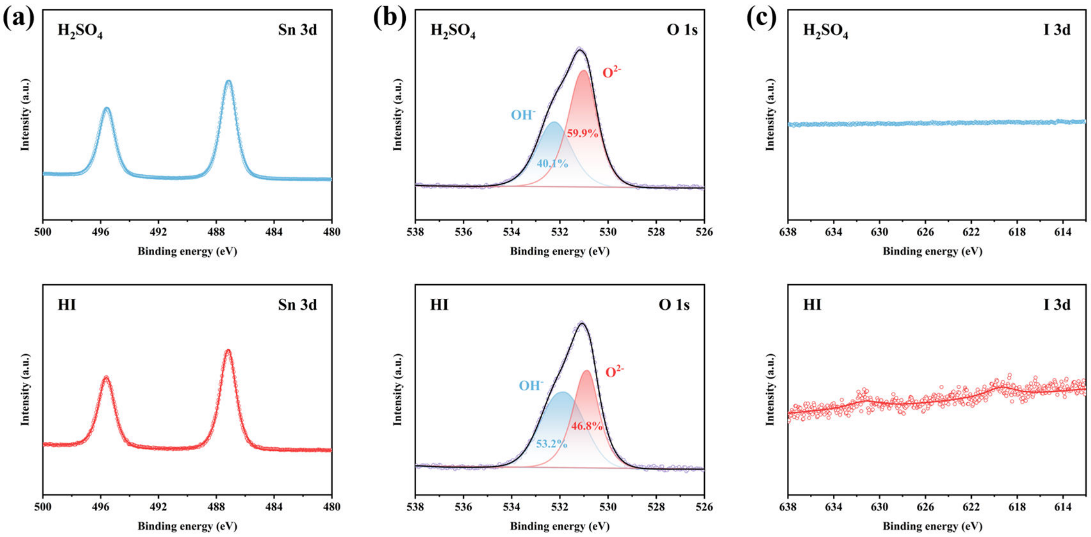

Figure S2 XPS spectra of SnO_2 films treated with H_2SO_4 and HI: (a) Sn 3d (b) O 1s (c) I 3d

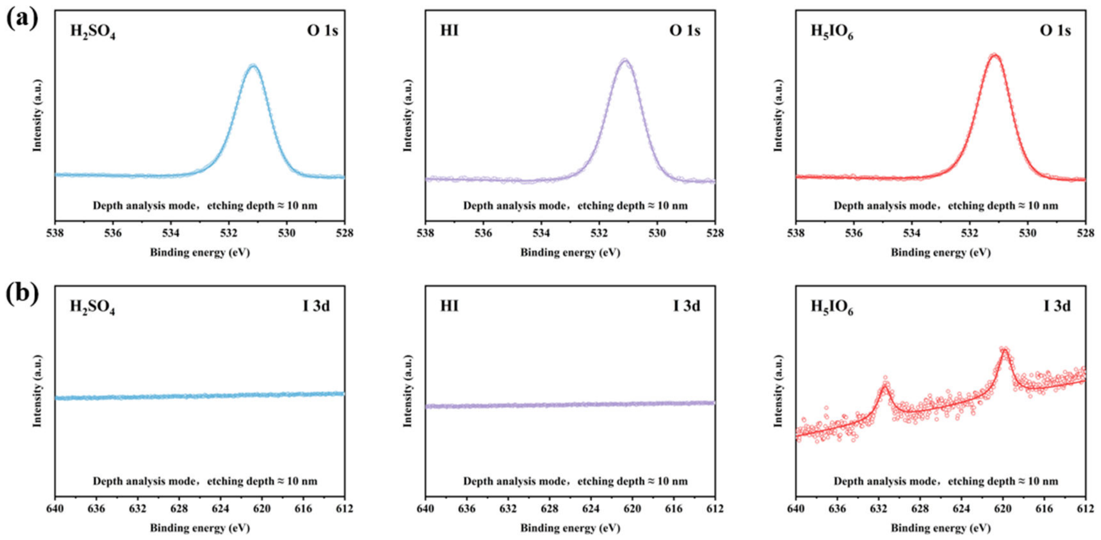

Figure S3 XPS spectra on depth analysis mode of SnO_2 films treated by several acids. (a) Sn 3d (b) I 3d

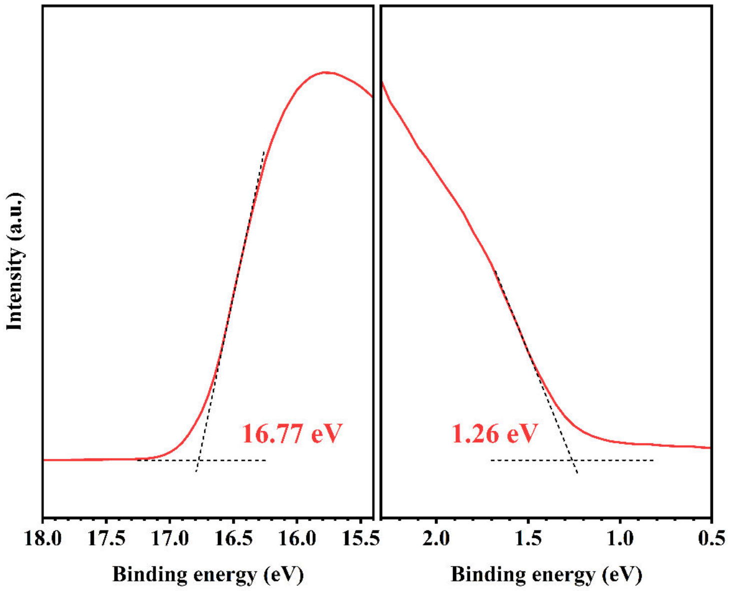

Figure S4 UPS spectrum of the corresponding perovskite film.

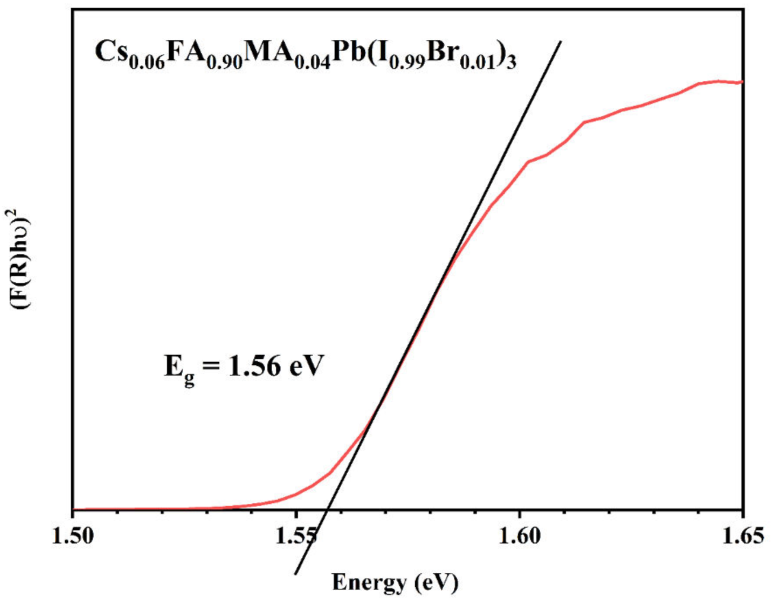

Figure S5 Optical bandgap calculated by Tauc plot of the perovskite films.

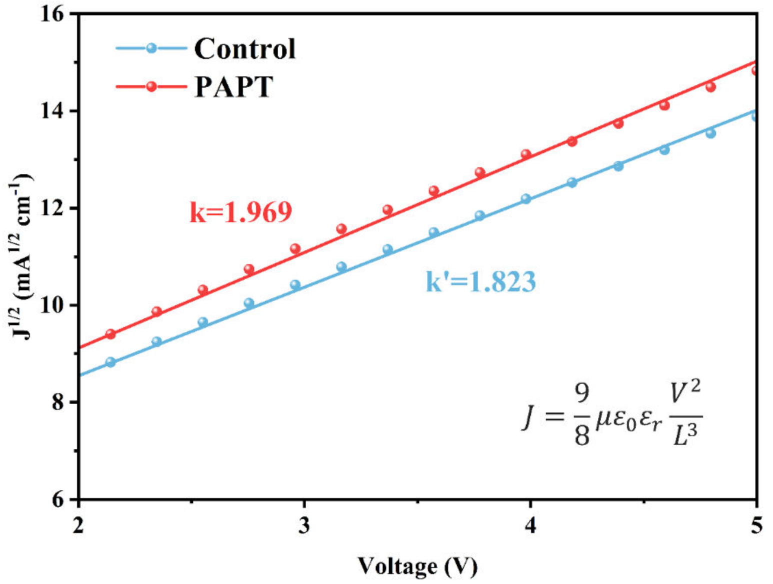

Figure S6 Mott-Gurney curves ($J^{1/2}$-V) for the calculation of electron mobility in pristine SnO_2 and PAPT-modified SnO_2 layer based on a device structure of FTO/ETL/Ag.

The electron mobility can be calculated by Mott-Gurney Law:

$$J = \frac{9}{8}\mu\varepsilon_0\varepsilon_r\frac{V^2}{L^3}$$

Where μ is free carrier mobility, ε_0 is permittivity of free space, ε_r is the dielectric

constant of the material, V is the applied voltage and L is the distance between electrodes.[8]

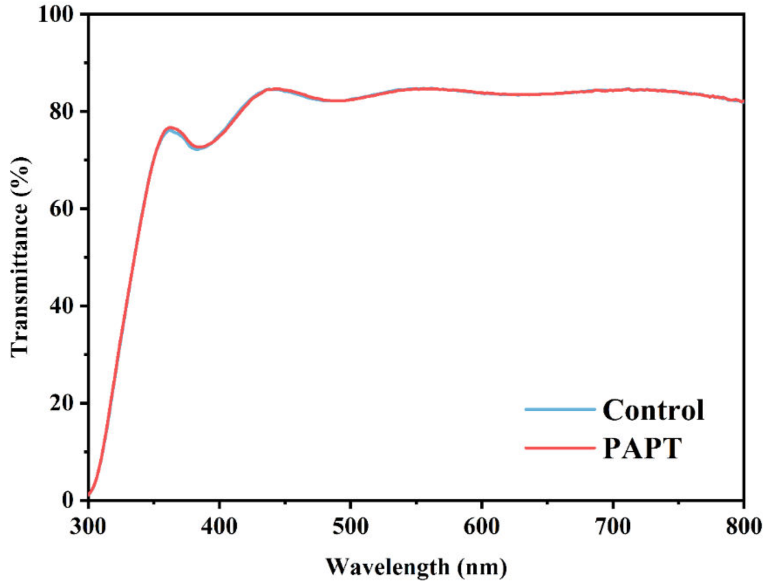

Figure S7 Transmittance spectra of control and PAPT-modified SnO_2 films.

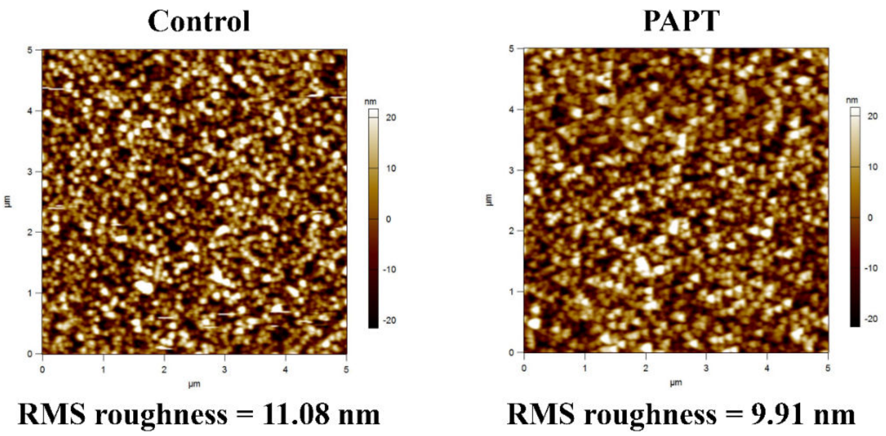

Figure S8 AFM images of pristine SnO_2 films and PAPT-modified SnO_2 films.

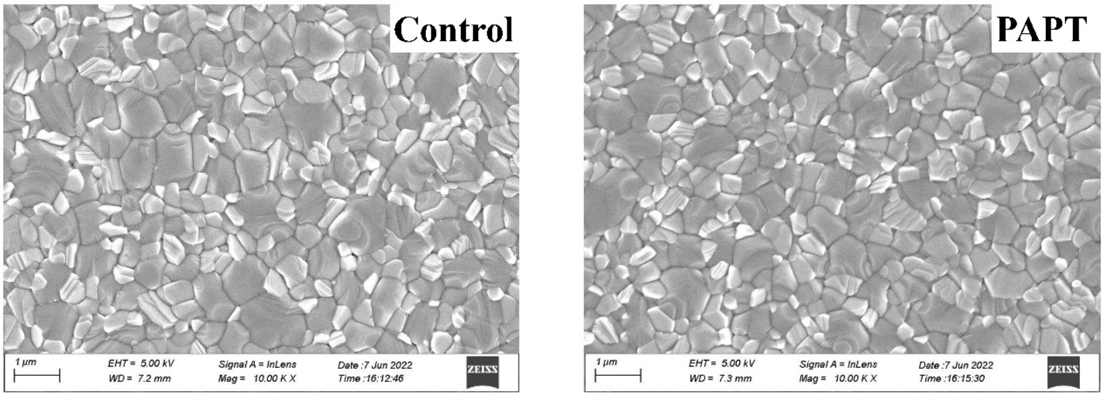

Figure S9 SEM image of perovskite films on control and PAPT-modified SnO_2 films.

Table S1 Fitting parameters of the bi-exponential decay function in TRPL spectra of the corresponding perovskite films deposited on the FTO/SnO_2 substrate using a 460 nm excitation light source.

Samples	***A1***	τ_1 **(ns)**	***A2***	τ_2 **(ns)**	τ_{avg} **(ns)**
**Control SnO_2**	0.48	12.42	0.52	146.92	82.70
**PAPT-modified SnO_2**	0.64	6.30	0.36	128.91	50.55

The average carrier lifetime (τ_{avg}) was calculated according to the equation:

$$\tau_{avg} = \frac{A_1\tau_1 + A_2\tau_2}{A_1 + A_2}$$

Table S2 R^2 value of the dark J-V characteristics of the electron-only devices.

Samples	Ohmic region	TFL region	SCLC region
**Control SnO_2**	0.9983	0.9997	0.9997
**PAPT-modified SnO_2**	0.9999	0.9987	0.9991

Table S3 The champion photovoltaic performance of PSCs with/without Phe addition

Conditions	J_{SC} (mA/cm^2)	V_{OC} (V)	*FF* (%)	PCE (%)
**Control**	25.09	1.06	74.28	19.77
**1 mM PAPT**	23.96	1.09	79.67	20.81
**2 mM PAPT**	25.02	1.09	81.55	22.25
**3 mM PAPT**	24.97	1.09	79.06	21.58
**4 mM PAPT**	24.91	1.06	78.47	20.77

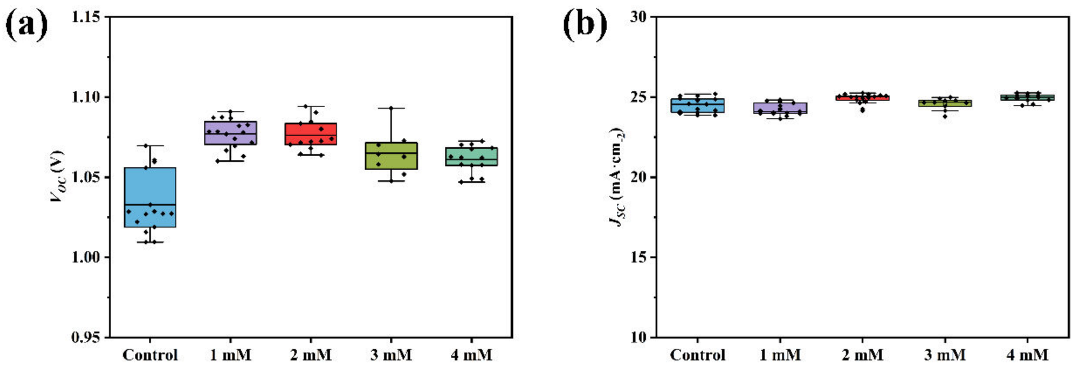

Figure S10 V_{OC} and J_{SC} statistic distribution of PSCs with different amounts of periodic acid treatment.

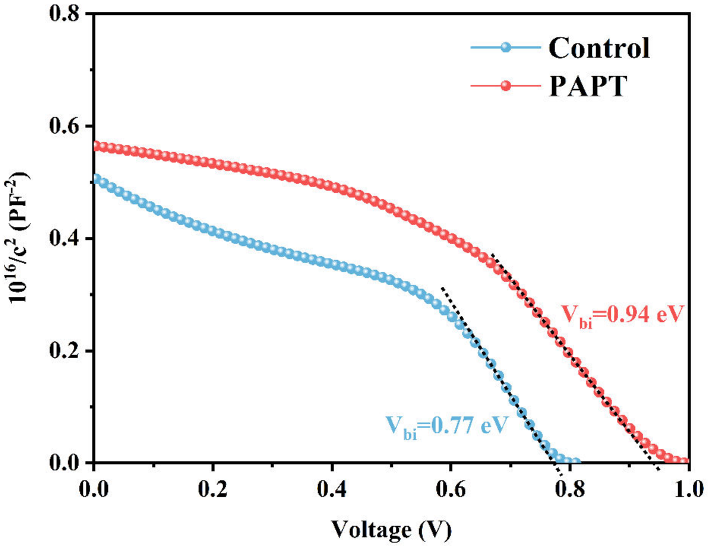

Figure S11 Electric capacity-voltage (*C-V*) measurement of the corresponding devices.

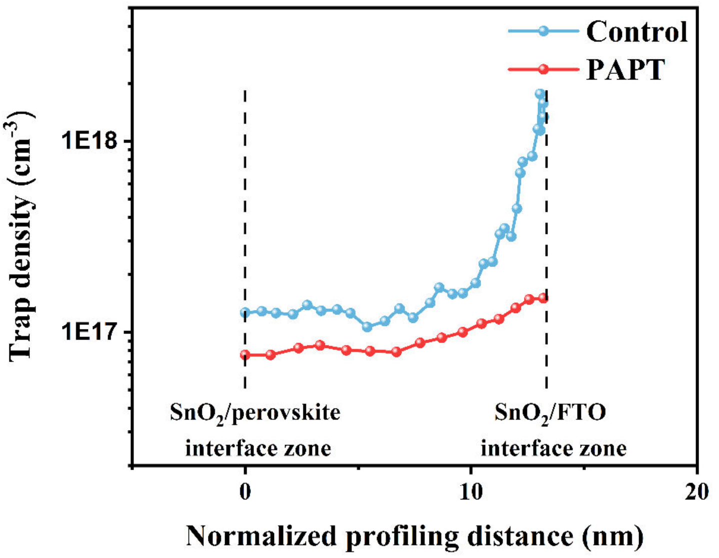

Figure S12 DLCP measurements of corresponding devices. Note: The x-axis has been adjusted to start from the SnO_2/perovskite interface.[9]

Table S4 EIS fitting results for PSCs based on different SnO_2 films under dark condition.

Samples	R_S (Ω)	R_{rec} (Ω)
Pristine SnO_2	29.49	240.6
PAPT-modified SnO_2	23.96	399.7

References

1. Liu, C., et al., *Tuning structural isomers of phenylenediammonium to afford efficient and stable perovskite solar cells and modules.* Nat Commun, 2021. **12**(1): p. 6394.
2. Kresse, G. and J. Furthmüller, *Efficient iterative schemes for ab initio total-energy calculations using a plane-wave basis set.* Physical Review B, 1996. **54**(16): p. 11169-11186.
3. Blöchl, P.E., *Projector augmented-wave method.* Physical Review B, 1994. **50**(24): p. 17953-17979.
4. Kresse, G. and D. Joubert, *From ultrasoft pseudopotentials to the projector augmented-wave method.* Physical Review B, 1999. **59**(3): p. 1758-1775.
5. Kresse, G. and J. Furthmüller, *Efficiency of ab-initio total energy calculations for metals and semiconductors using a plane-wave basis set.* Computational Materials Science, 1996. **6**(1): p. 15-50.

6. Ernzerhof, M. and G.E. Scuseria, *Assessment of the Perdew–Burke–Ernzerhof exchange-correlation functional.* The Journal of Chemical Physics, 1999. **110**(11): p. 5029-5036.
7. Grimme, S., et al., *A consistent and accurate ab initio parametrization of density functional dispersion correction (DFT-D) for the 94 elements H-Pu.* The Journal of Chemical Physics, 2010. **132**(15): p. 154104.
8. Murgatroyd, P., *Theory of space-charge-limited current enhanced by Frenkel effect.* Journal of Physics D: Applied Physics, 1970. **3**(2): p. 151.
9. Xiong, Q., et al., *$NdCl_3$ Dose as a Universal Approach for High-Efficiency Perovskite Solar Cells Based on Low-Temperature-Processed SnOx.* ACS Applied Materials & Interfaces, 2020. **12**(41): p. 46306-46316.
